# Sequence Diversity in tRNA Gene Locus A-L among Iranian Isolates of *Entamoeba dispar*

**Published:** 2012

**Authors:** E Nazemalhosseini-Mojarad, M Azimirad, Z Nochi, S Romani, M Tajbakhsh, M Rostami-Nejad, A Haghighi, MR Zali

**Affiliations:** 1 Research Institute for Gastroenterology and Liver Diseases, Shahid Beheshti University of Medical Sciences, Tehran, Iran; 2 Department of Medical Parasitology & Mycology, School of Medicine, Shahid Beheshti University of Medical Sciences, Tehran, Iran

**Keywords:** *Entamoeba dispar*, STRs, Locus A-L, Diversity, Iran

## Abstract

**Background:**

A number of methods for detecting diversity in *Entamoeba* have been described over the years. In the present study the genetic polymorphism of noncoding locus A-L was analyzed using PCR and sequencing in order to clarify the genotypic differences among *E. dispar* isolates.

**Methods:**

A total of 28 *E. dispar* from patients with gastrointestinal symptoms were determined and the genomic DNA was extracted directly from stool. For genotype analysis; Locus A-L was amplified by PCR and PCR products were sequenced. The sequences obtained were edited manually and aligned using Gene Runner software.

**Results:**

With sequencing of PCR products a reliable genetic diversity in size, number and position of the repeat units were observed among the Iranian *E. dispar* isolates in locus A-L gene. Sequences showed variation in length from 448bp to 507bp and seven distinct types were identified.

**Conclusion:**

The genetic diversity of loci like A-L shows them to be suitable for epidemiological studies such as the characterization of the routes of transmission of these parasites in Iran.

## Introduction

A number of methods for detecting the diversity in *Entamoeba* have been described over the years (1), but recently Ali et al. investigated the association between the genotypes of parasites and the clinical outcome of infection (2) using a 6-locus genotyping system based on tRNA-linked short tandem repeats (STRs) (3). The six targets for amplification in this method (Loci S-Q, D-A, A-L, S^TGA^-D, R-R and N-K2) were selected from among over 40 STR-containing loci linked to tRNA genes in *E. histolytica* (1). One of these polymorphic loci is Locus D-A, previously called locus 1-2, has been shown to be potentially useful for investigating the molecular epidemiology of *E. histolytica* and *E. dispar* (4, 5).

Zaki et al. isolated and characterized locus D-A, and later compared the nucleotide sequence of this locus between *E. dispar* and *E. histolytica*. This revealed significant differences in both the STRs and the flanking regions (6, 7). Haghighi et al. analyzed the genetic polymorphisms of four loci, including D-A, 5-6 among 79 isolates of *E. histolytica* obtained from different geographic regions. They reported large scale genetic differentiation between Japanese and Thai isolates (4, 8). Recently, molecular studies have been extended to distinguish and investigate the distribution of these two species in Iran (9–11).

In the present study, genetic polymorphism of another tRNA-liked STR-containing locus, A-L, was analyzed using PCR and sequencing methods in order to clarify further the genotypic differences among *E. dispar* isolates.

## Materials and Methods

A total of 28 *E. dispar* strains were analyzed. Twenty four of them were collected from Iranian patients referred to the clinical laboratories of hospitals in the city of Tehran and Zahedan and 4 strains were collected from asymptomatic individuals referred to health care centers in the city of Gonbad (5). Clinical information on the samples is given in Table 1. All the samples used in this study were diagnosed as positive for *Entamoeba* spp. by microscopic examination of fresh stools using direct smears, formalin-ether concentrated, and trichrome stained specimens.

**Table 1:** Background and genotype of *E. dispar* isolates

No.	Isolates	Isolation		Clinical symptoms^a^	Sex	Age (yr)	Size of PCR fragments (bp)	Accession numbers	Type Locus A-L	Type Locus D-A (5)
		date	location							
1	NH1IR	2006	Tehran	Abdominal pain, diarrhea	F	20	449	HQ439931	F	IV
2	NH2IR	2006	Tehran	Abdominal pain	M	6	449	HQ439932	F	VII
										
3	NH3IR	2006	Tehran	Abdominal pain, bloating	M	22	449	HQ439933	F	II
4	NH4IR	2006	Tehran	Abdominal pain	M	32	499	HQ439934	B	I
5	NH5IR	2006	Tehran	Abdominal pain, vomiting	F	27	507	HQ439935	A	I
6	NH6IR	2006	Tehran	Abdominal pain	M	63	472	HQ439936	E	VI
7	NH7IR	2007	Tehran	Diarrhea, bloating	M	33	483	HQ439937	C	IV
8	NH8IR	2007	Tehran	Abdominal pain, diarrhea	F	24	483	HQ439938	C	III
9	NH9IR	2007	Tehran	diarrhea	F	36	499	HQ439939	B	VI
10	NH10IR	2007	Tehran	Abdominal pain	F	38	507	HQ439940	A	III
11	NH11IR	2007	Tehran	Abdominal pain, bloating	F	63	499	HQ439941	B	III
12	NH12IR	2007	Tehran	Abdominal pain	M	64	483	HQ439942	C	X
13	NH13IR	2007	Tehran	Abdominal pain	M	42	507	HQ439943	A	V
14	NH14IR	2007	Tehran	Abdominal pain, vomiting	M	54	483	HQ439944	D	I
15	NH15IR	2007	Tehran	Abdominal pain	M	53	472	HQ439945	E	X
16	NH16IR	2007	Tehran	Abdominal pain, bloating	F	8	448	HQ439946	G	IV
17	NH17IR	2007	Tehran	Diarrhea, vomiting	M	14	472	HQ439947	E	I
										
18	NH18IR	2007	Tehran	Abdominal pain	F	12	472	HQ439948	E	III
19	NH19IR	2007	Tehran	Abdominal pain, vomiting	F	20	499	HQ439949	B	X
20	NH20IR	2007	Tehran	Abdominal pain, diarrhea	F	31	499	HQ439950	B	X
21	NH21IR	2007	Tehran	Abdominal pain, diarrhea	F	8	507	HQ439951	A	X
22	SHN3IR	2004	Zahedan	Abdominal pain	F	25	472	HQ439956	E	IX
23	SHN4IR	2004	Zahedan	Abdominal pain, vomiting	M	42	472	HQ439957	E	IX
24	SHN7IR	2004	Zahedan	Abdominal pain, vomiting	M	32	472	HQ439958	E	XII
25	NHM1IR	2005	Gonbad	Asymptomatic	F	28	472	HQ439952	E	XI
26	NHM2IR	2005	Gonbad	Asymptomatic	M	31	472	HQ439953	E	VII
27	NHM3IR	2005	Gonbad	Asymptomatic	M	31	472	HQ439954	E	VII
28	NHM4IR	2005	Gonbad	Asymptomatic	M	31	472	HQ439955	E	XI

The genomic DNA was extracted directly from stool and samples were identified to species level by locus D-A based PCR analysis, as previously described (5). For genotype analysis, Locus A-L was amplified by PCR with the primer set 5′-CATCTCCAT TATTATGTATCTATTTATCTATTTA-3′and 5′- GGCACGAATGCTTTGATATATAA-3′ (3). PCR products were analyzed by electrophoresis using 1.8% agarose gels (Fermentas, #R0491) in Tris-boric acid-EDTA buffer containing ethidium bromide after which the gels were photographed under ultraviolet light (UVIdoc, UVItec Limited, Cambridge, United Kingdom). The PCR products were sequenced using the amplification primers and an Applied Biosystems (ABI) Terminator Cycle Sequencing Ready Reaction kit (BigDye^®^ Terminator V3.1 Cycle Sequencing Kit) on an ABI 3130xl Genetic Analyzer. The sequences obtained were edited manually and aligned using Gene Runner software (version 3.05). Nuc-leotide sequences, except forward and reverse primer regions, were aligned with the only previously available locus A-L sequence from *E. dispar* in GenBank (AY842969). All sequences were submitted to the GenBank/EMBL/DDBJ database under accession numbers *HQ439931- HQ439958*.

## Results

**Fig. 1:**
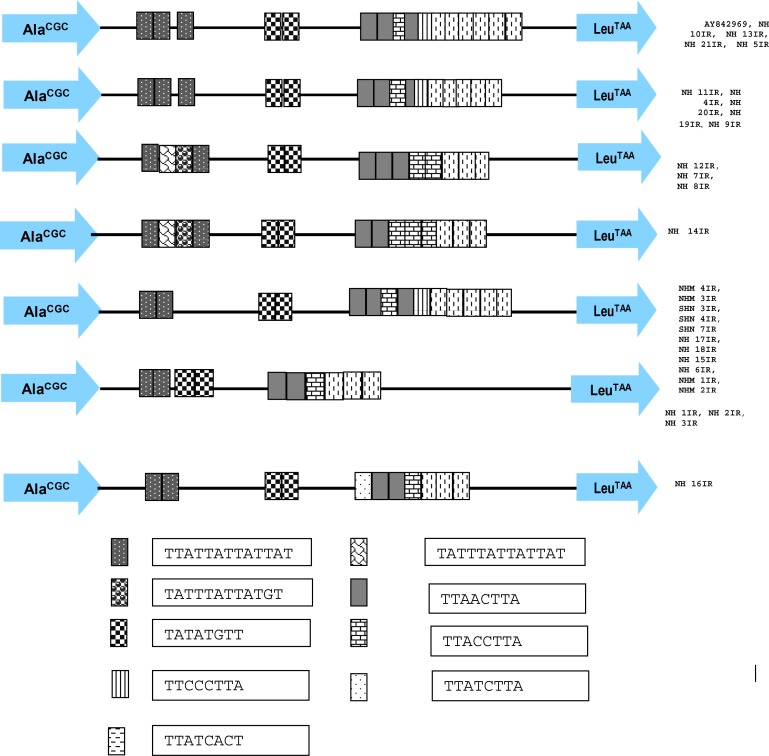
Schematic representation of the STR polymorphisms in locus A-L of *E. dispar*. The 7 distinct sequence types are shown as well as the identification tag for the isolates that matched each type; also shown is the structure of locus A-L sequence in the standard isolate, *E. dispar* SAW760 (AY842969). The sequences of each of the nine repeat types are shown beside their corresponding colored block. The conserved non-repeated regions are shown as a single line

In order to investigate genetic diversity, the PCR-amplified products from 28 *E. dispar* isolates were subjected to direct sequencing. Samples were sequenced in both directions and, when any variations were found, results were confirmed by sequencing of at least two independent PCR products. All sequences were analyzed by Chromas version 1.45 (Technelysium, Queensland, Australia) and the sequence homology was compared with the sequences in GenBank by BLAST analysis. PCR amplification and sequencing of the PCR products showed a remarkable level of genetic diversity in size, number and position of the repeat units among the *E. dispar* isolates (Fig. 1). Nucleotide sequence length varied from 448bp to 507bp which led to visible differences in PCR product size (Table 1). Seven distinct nucleotide sequences were obtained from the isolates while gel analysis of the PCR products show three groups distinguishable by size. Sequence E represents the dominant genotype (11/28, 37%) among the Iranian isolates and its 472 bp fragment was also the most frequent size found. The STR organization in locus A–L from *E. dispar* SAW760 (AY842969), which has 507 nucleotides, is compared to the organization in sequences from the Iranian isolates in Fig. 1.

## Discussion

The ability to identify strains of *Entamoeba dispar* may lead to insights into the population structure and epidemiology of the organism. When polymorphism in two *E. histolytica* loci, 1-2 and 5-6, was studied in 2001 by Zaki et al., the remarkable diversity in length, type and numbers of the repeat units found showed that they have the potential to allow the investigation of genetic differences between invasive and noninvasive *E. histolytica* isolates. Sequences corresponding to the polymorphic loci reported from *E. histolytica* have also been detected in *E. dispar*. Comparison of nucleotide sequences in two loci between *E. dispar* and *E. histolytica* revealed significant differences in both the repeats and the flanking regions, which allowed the typing and differentiation of these two parasites simultaneously (6, 7). However although variation in locus A-L has been investigated to some extent in *E. histolytica* (12), it has not been investigated previously in *E. dispar*. The tRNA gene regions in locus A-L are conserved and are the site of the primers used, but in the middle there are repeat units of between 8 and 15 nucleotides which vary among isolates. Elimination, duplication and sub-stitution of units in this repeat-containing region are the basis of polymorphisms detected in the two species.

In our previous study PCR amplification of locus D-A among Iranian *E. dispar* isolates, showed a remarkable genetic diversity in size and this result confirmed by Sequencing of PCR products (5). By simultaneous investigation of locus A-L and locus D-A (5), 26 subtypes out of 28 *Entamoeba dispar* isolates were distinguished (the molecular patterns of NH19IR and NH20IR, also NHM2IR and NHM3IR are not different in two loci) (Table 1).

In this study, no meaningful correlation between infection with *E. dispar* and age, sex or parasite genotype was observed. However, it appears that sequence type E is over-represented in the male individuals compared to females (out of 11 individuals who showed this type, 8 of these were males) or sequence type E is common in asymptomatic patients. In 2001, in Bangladesh, the role of genetic diversity in *E. histolytica* virulence was studied and it was clarified that the genetic diversity of *E. histolytica* subspecies in endemic regions is because of SREHP polymorphism. Noticeably, the polymorphism of liver amebiasis subspecies was different from intestinal amebiasis subspecies (13–14).

Haghighi et al. reported a considerable polymorphic in size, number and position of the repeat units in four loci (1-2, 5-6, SREHP and Chitinase) of different *E. histolytica* isolates obtained from stool samples of mentally handicapped individuals and male homosexuals from different regions of Japan (4, 8). They proposed that genotyping of ameba isolates should help to determine geographic origins of isolates and routes of transmission. Although the studies of Haghighi et al. did not detect a link between genotype and symptoms, their samples were from geographically diverse sources and acquired over a number of years (4, 8).

The studies of Ali et al. developed a reliable method for PCR-based genotyping of *E. histolytica* based on variation in the numbers of short tandem repeats that are linked to tRNA genes in this species and suggesting that the parasite genome does contribute in some way to the outcome of infection with *E. histolytica* (2, 3).

In conclusion, we propose that molecular typing and analysis of genotypes of *E. histolytica* and *E. dispar* isolates from a variety of locations should help in determining the geographic origins of isolates and routes of transmission.

## References

[1] Clark CG (2006). Methods for Investigation of Diversity in *Entamoeba histolytica*. Arch Med Res..

[2] Ali IK, Mondal U, Roy S, Haque R, Petri WA, Clark CG (2007). Evidence for a Link between Parasite Genotype and Outcome of Infection with *Entamoeba histolytica*. J Clin Microbiol..

[3] Ali IK, Zaki M, Clark CG (2005). Use of PCR amplification of tRNA gene-linked short tandem repeats for genotyping *Entamoeba histolytica*. J Clin Microbiol..

[4] Haghighi A, Kobayashi S, Takeuchi T, Masuda G, Nozaki T (2002). Remarkable genetic polymorphism among *Entamoeba histolytica* isolates from a limited geographic area. J Clin Microbiol.

[5] Nazemalhosseini- Mojarad E, Haghighi A, Kazemi B, Rostami -Nejd M, Abadi A, Zali MR (2009). High genetic diversity among Iranian *Entamoeba dispar* isolates based on the noncoding short tandem repeat locus D-A. Acta Trop.

[6] Zaki M, Clark CG (2001). Isolation and characterization of polymorphic DNA from *Entamoeba histolytica*. J Clin Microbiol.

[7] Zaki M, Meelu P, Sun W, Clark CG (2002). Simultaneous differentiation and typing of *Entamoeba histolytica and Entamoeba dispar*. J Clin Microbiol.

[8] Haghighi A, Kobayashi S, Takeuchi T, Thammalerd N, Nozaki T (2003). Geographic diversity among genotypes of *Entamoeba histolytica* field isolates. J Clin Microbiol..

[9] Hooshyar H, Rezaian M, Kazemi B, Jeddi-Tehrani M, Solaymani-Mohammadi S (2004). The distribution of *Entamoeba histolytica and Entamoeba dispar* in northern, central, and southern Iran. Parasitol Res..

[10] Solaymani-Mohammadi S, Rezaian M, Babaei Z (2006). Comparison of a stool antigen detection kit and PCR for diagnosis of *Entamoeba histolytica* and *Entamoeba dispar* infections in asymptomatic cyst passers in Iran. J Clin Microbiol.

[11] Nazemalhosseini- Mojarad E, Nochi Z, Sahebekhtiari N (2010). Discrimination of *Entamoeba mos-hkovskii* in patients with gastrointestinal disorders by single-round PCR. Jpn J Infect Dis.

[12] Escueta-Decadiza A, Kobayashi S, Takeuchi T, Tachibana H, Nozaki T (2010). Identification of an avirulent *Entamoeba histolytica* strain with unique tRNA-linked short tandem repeat markers. Parasitol Int..

[13] Ayeh-Kumi PF, Ali IK, Lockhart LA, Gilchrist CA, Petri WA, Haque R (2001). *Entamoeba histolytica*: genetic diversity of clinical isolates from Bangladesh as demonstrated by polymorphisms in the serine-rich gene. Exp Parasitol..

[14] Simonishvili S, Tsanava S, Sanadze K, Chilikadze R, Miskalishvili A, Lomkatsi N, Imnadze P, Petri WA, Trapaidez N (2005). *Entamoeba histolytica*: the serine-rich gene polymorphism-based genetic variability of clinical isolates from Georgia. Exp Parasitol..

